# Epidemiology and risk factors of respiratory syncytial virus associated acute respiratory tract infection in hospitalized children younger than 5 years from Sri Lanka

**DOI:** 10.3389/fmicb.2023.1173842

**Published:** 2023-06-26

**Authors:** Maduja V. M. Divarathna, Rukshan A. M. Rafeek, Adrian J. Morel, Chathuri Aththanayake, Faseeha Noordeen

**Affiliations:** ^1^Department of Microbiology, Faculty of Medicine, Diagnostic and Research Virology Laboratory, University of Peradeniya, Peradeniya, Sri Lanka; ^2^General Hospital-Kegalle, Kegalle, Sri Lanka; ^3^Faculty of Management, University of Peradeniya, Peradeniya, Sri Lanka

**Keywords:** respiratory syncytial virus, acute respiratory tract infections, epidemiology, risk factors, children, Sri Lanka

## Abstract

**Background:**

Respiratory syncytial virus (RSV) is the leading cause of acute respiratory tract infections (ARTI) and a major cause of morbidity and mortality in children worldwide.

**Aim:**

This study aimed to describe the prevalence and seasonal patterns of RSV and to determine the actual and predictive association of RSV-associated ARTI and clinical, socio-demographic, and climatic risk factors in children < 5 years.

**Methods:**

Nasopharyngeal aspirates were collected from 500 children < 5 years admitted to the Kegalle General Hospital, Sri Lanka between May 2016 to July 2018. RSV and RSV subtypes were detected using immunofluorescence assay and real time RT-PCR, respectively. Descriptive and inferential statistics were done for the data analysis using Chi-square, Fisher’s exact, Kruskal–Wallis test, and multiple binary logistic regression in the statistical package for social sciences (SPSS), version 16.0.

**Results:**

Prevalence of RSV-associated ARTI was 28% in children < 5 years. Both RSV subtypes were detected throughout the study period. RSV-B was the dominant subtype detected with a prevalence of 72.14%. RSV infection in general caused severe respiratory disease leading to hypoxemia. Compared to RSV-B, RSV-A infection had more symptoms leading to hypoxemia. Factors increasing the risk of contracting RSV infection included number of people living (*n* > 6), having pets at home and inhaling toxic fumes. The inferential analysis predicts RSV infection in children < 5 years with ARTI, with a 75.4% probability with clinical and socio-demographic characteristics like age < 1 year, fever for > 4 days, cough, conjunctivitis, stuffiness, fatigue, six or more people at home, having pets at home and inhaling toxic fumes. Climatic factors like increases in temperature (°C), wind speed (Km/h), wind gust (Km/h), rainfall (mm) and atmospheric pressure (mb) showed a strong correlation with the RSV infection in children.

## 1. Introduction

Respiratory syncytial virus (RSV) has been identified as a leading cause of acute respiratory tract infection (ARTI) in infants and children worldwide (Shi et al., 2017). High rates of hospitalization of children with RSV-associated ARTI cause a substantial burden to the health care systems in low and middle-income countries (LMICs; Srikantiah et al., 2021). According to the estimates made in a global burden study conducted in 2019, in children younger than 5 years, 33 million episodes of RSV-associated ARTI are reported with 3.6 million hospitalizations and 26,300 in-hospital deaths. Furthermore, 1.4 million episodes of RSV-associated ARTI caused 13,300 in-hospital deaths in infants. Overall mortality of RSV-associated ARTI is estimated to be as high as 45,700, which is 2% of the mortality occurring in children younger than 1 year and 3.6% of the mortality occurring in children younger than 6 months. Moreover, more than 95% of all RSV-associated ARTI episodes and more than 97% of the RSV-attributable deaths in children occurred in LMICs (Li et al., 2022).

RSV outbreaks have periodic emergence patterns (Weber et al., 1998). In temperate regions, RSV-activity positively correlates with high relative humidity and low temperature (Bloom-Feshbach et al., 2013). In tropical regions, RSV outbreaks occur mostly in wet seasons with seasonal rainfall and RSV-activity peaks after the onset of seasonal rains. Countries located closer to the equator with perennial high rainfall and large islands show a distinct pattern of RSV-activity, which is present throughout the year or half of the year (Berman et al., 1983; Doraisingham and Ling, 1986; Weber et al., 1998).

Most children infected with RSV may have mild upper respiratory tract symptoms like runny nose, cough and wheezing. However, RSV infection can escalate into more adverse symptoms like bronchiolitis and pneumonia (Shi et al., 2017). RSV infection in children has a remarkable variability in severity, ranging from insignificant clinical illness to severe respiratory distress (Houben et al., 2010). According to the classification of World Health Organization, severe and very severe ARTI in children < 5 years age was, respectively, hypothesized as having breathing difficulties, which leads to hypoxemia and requiring intensive care (Roth et al., 2015; Fitzner et al., 2018). Many studies have identified an association between RSV subtypes with severe disease over the years but the association was identified with different subtypes in different studies. However, linking a specific RSV subtype to severe disease appears challenging due to lack of consistency in supportive data. Consequently, further research is necessary to learn more about the RSV subtype differences in disease severity (Fletcher et al., 1997; Vandini et al., 2017).

Factors predisposing to acquire RSV infection in previously healthy children appear ambiguous and are likely to be determined by the host, environment and viral characteristics (Collins and Graham, 2008). Host derived risk factors for RSV infection include prematurity, low birth weight, young age (<6 months), male sex, ethnicity, congenital heart disease, broncho-pulmonary dysplasia, cardiopulmonary disease, chronic lung disease, cystic fibrosis, Down syndrome, atopy, family history of asthma and compromised immunity (Simoes, 2003; Nagayama et al., 2006; Stein et al., 2017). Environmental risk factors associated with RSV infection include crowded living conditions, low socioeconomic status, indoor air pollution, exposure to passive smoking, day care attendance, breast feeding, living at high altitude and month of birth in relation to RSV seasonality (Simoes, 2003; Stensballe et al., 2003). The contribution of direct viral cytopathology to the pathogenesis of RSV infection remains controversial (Collins and Graham, 2008). However, some data indicate that there is a difference in the pathogenicity between RSV-A and RSV-B subtypes (Hall et al., 1990; Laham et al., 2017).

The current study was conducted for 26 months in 500 hospitalized children < 5 years of age. This study aimed to identify the association between disease severity of RSV-associated ARTI and clinical, socio-demographic, climatic-risk factors. This is the first Sri Lankan study to emphasize the impact of RSV subtypes. Moreover, the association between risk factors and disease severity of RSV-associated ARTI in children in developing countries like Sri Lanka is not well defined. This study has done a comprehensive analysis on RSV epidemiology generating new knowledge on RSV infection in children < 5 years in Sri Lanka.

## 2. Materials and methods

### 2.1. Study design and study population

The ethical approval (Permit No: 2016/EC/91) for the study was obtained from the Ethical Review Committee of the Faculty of Medicine, University of Peradeniya, Sri Lanka (Supplement 1). A total of 500 nasopharyngeal aspirate (NPA) samples were collected from children < 5 years hospitalized between May 2016 and July 2018 at the General Hospital, Kegalle, which is a major state hospital in the Kegalle district of Sri Lanka. Children hospitalized with a history of ARTI of <4 days, recurrent RTI and hereditary or anatomical anomalies in cardiovascular and respiratory systems with ARTI were included in the study. Children hospitalized with ARTI between < 1 month and > 5 years, suspected or established bacterial RTI and children not consenting for collecting NPA were excluded from the study.

The WHO classification of severe ARTIs is based on different age groups such as infants < 2 months, infants and children between 2 months and 5 years and individuals above 5 years. Among children aged between 2 months and 5 years, the characteristics used to identify severe ARTIs are cough or difficulty breathing and a breathing rate above 50 and 40 breaths. Among infants aged < 2 months, the characteristics used to identify severe ARTIs are cough or difficulty breathing, requiring hospitalization and showing at least one of five danger signs (unable to drink or breastfeed, vomiting when ingested, having convolution, being lethargic or unconscious, showing signs of chest in-drawing; Roth et al., 2015; Fitzner et al., 2018).

The classification and case definitions for severe ARTIs in the current study were based on the WHO classification. The study strictly adhered to the criteria of breathing difficulties leading up to hypoxemia in children between 2 months and 5 years as the study sample consist of infants and children between 1 month and 5 years. ARTIs were defined as mild, moderate, severe and very severe by the pediatrician during the clinical assessment-1. Mild ARTI—cough without fast breathing, without chest in-drawing, blocked or runny nose and sore throat; 2. Moderate ARTI—cough with fast breathing and chest in-drawing; 3. Severe ARTI—ARTI with hypoxemia with dyspnea (labored breathing) or tachypnoea (abnormally rapid breathing) or shortness of breath or difficulty in breathing; 4. Very severe ARTI—ARTI requiring high dependency or intensive care. The same classification/case definition for severe ARTIs has been used in a recent global study on RSV burden in children younger than 5 years (Li et al., 2022). Moreover, in the current study, severity of bronchiolitis is classified into mild, moderate and severe based on a modified assessment used by New Zealand guidelines and Scottish Intercollegiate Guidelines Network guidelines (Øymar et al., 2014).

### 2.2. Sample collection and sample processing

A written informed consent was obtained from parents or guardians of children enrolled in the study prior to sample collection. The NPA samples were collected within 48 h of admission by the collaborating pediatrician using a recommended mucus extractor (Pacific Hospital Supply Co., Ltd., Taiwan) and 0.9% saline as the virus transport medium. NPA samples were directly diluted in phosphate buffered saline, and then processed by multiple centrifugation and vortexing steps until the cell sediment was formed.

Then the specimen for direct testing was prepared by adding 1,000 μL of phosphate buffered saline to re-suspend the cell pallet. The NPA samples were stored at 4°C for less than 24 h for antigen detection. After completing the antigen detection by an immunofluorescence assay (IFA), the rest of the NPAs were stored at-80°C until processed for RNA extraction and real time PCR testing for RSV subtyping.

### 2.3. Detection of RSV using IFA and RT-PCR

Antigen detection for RSV in NPA was done by IFA (D3 Ultra Respiratory Virus Screening and ID Kit—Diagnostic Hybrids, United States—Catalog No: 01-010000.v2) along with seven viruses: RSV, influenza-A (Inf-A), Inf-B, adenovirus, para influenza-1 (PIV-1), PIV-2, and PIV-3. Fluorescence microscopy for IFA was done under Leitz Diaplan and Zeiss Axio-cam fluorescent microscopes, Germany for the detection of cells expressing specific antigens for these seven viruses with positive and negative controls. Detailed findings on Inf-A, Inf-B and PIV-1, PIV-2 and PIV-3 are presented in two research articles published from the same program (Rafeek et al., 2021, 2022).

Nucleic acid extraction from IFA positive samples for RSV was conducted using QIAamp Viral RNA Mini Kit (Qiagen, Germany, Catalog No: 52906). Extracted RNA were tested by a real-time reverse transcription polymerase chain reaction (rtRT-PCR) for RSV subtyping (RealStar^®^ RSV RT-PCR Kit 3.0 Altona Diagnostics, Germany, Catalog No: 193013), using the Rotor-Gene 6,000 real time PCR machine and Rotor-Gene Q Series Software 2.3.1 (Corbett Life Science, Australia).

### 2.4. Clinical, socio-demographic and climatic data collection

Clinical and socio-demographic data (clinical diagnosis, symptoms, age, sex, ethnicity, residency and a wide range of potential social, environmental, and health risk factors) were obtained using a pre-tested questionnaire. Average rain fall (mm) data of Kegalle region within the study period was obtained from Department of Meteorology, Sri Lanka. Other data such as average temperature (°C), average humidity (%), average number of rainy days (n), average wind speed (Km/h) and average wind gust (Km/h) of Kegalle region within the study period was obtained from World Weather Online—application programming interface (Terence et al., 2015).

### 2.5. Statistical data analysis

Descriptive and predictive statistical analyses were carried out to identify the key features associated with the RSV infected children in this study. Data were double-checked and entered into a spreadsheet prepared in Microsoft^®^ Excel 2013 and statistical analysis was done using the Statistical Package for Social Sciences (SPSS), version 16.0. Association between the RSV prevalence and independent variables were analyzed using Kruskal-Wallis test (continuous variables) and chi-square test (categorical variables). Analysis for small sized samples was conducted using Fisher’s Exact Test. Multiple binary logistic regression analysis was used to do a predictive analysis of associations between a positive test for RSV with general variables. Correlations between climatic factors and viral positivity were analyzed using Spearman’s correlation. *p* value of < 0.05 was considered as statistically significant for all tests.

## 3. Results

### 3.1. Detection of RSV and RSV subtypes

A total of 237 (47.4%) of the 500 children were positive for the respiratory viruses including RSV by IFA. RSV was the most common respiratory virus detected compared to other viruses and most of the co-infections were associated with RSV. Overall, 140 of the 237 respiratory viruses’ positive children (59.07%) were positive only for RSV and the rest were positive for other 6 respiratory viruses tested. A total of 28 (11.81%) children were co-infected and of that, 27 children had dual and 1 child had a triple viral co-infection. Twenty four out of the 28 co-infections were associated with RSV (85.71%), 13 children had PIV-3 and RSV, 5 children had Inf-B and RSV, 3 children had Inf-A and RSV, 2 children had adenovirus and RSV and 1 child had Inf-A and Inf-B and RSV. Overall, a total of 164 RSV infections were detected in the current study sample including mono-and co-infections. RSV-B was the most dominant subtype detected (101/164, 61.58%), 46/164 (28.04%) children were infected with RSV-A and 17/164 (10.36%) children were co-infected with RSV-A and B.

### 3.2. Seasonal distribution of RSV infection

**Figure 1:**
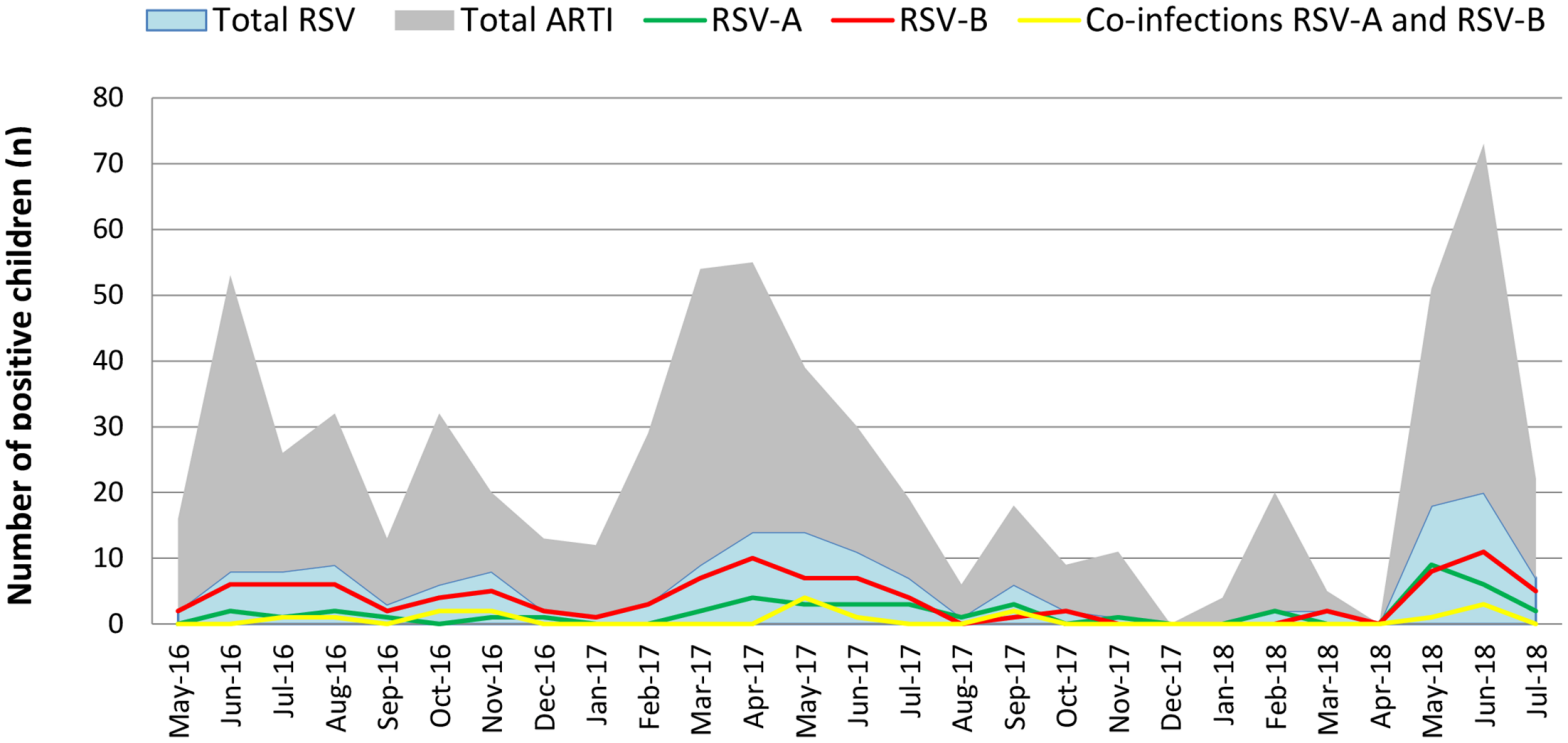
Monthly distribution of the RSV-A and RSV subtypes during the study period. RSV-B was detected throughout the study period with peak incidence from June to August 2016, March to June 2017 and April to June 2018. RSV-A was also detected throughout the year with a lesser incidence than RSV-B. RSV-A peaked from March to July 2017 and April to June 2018. RSV-A incidence during the 2018 outbreak (April–June 2018) was significantly higher compared to the 2016 and 2017 outbreaks. In 2018, the RSV-A incidence was more or less like the RSV-B incidence reported in that year. Co-infections between RSV-A and RSV-B were detected during the study period.

RSV was prevalent throughout the year with peaks in certain months. Major RSV peaks were observed from June to August 2016, March to July 2017 and May to July 2018. Minor RSV peaks were observed from October to November 2016 and September 2017. RSV positivity increased in peak periods every year and RSV was prevalent throughout the study period. Even though, RSV-B showed a dominant circulation, RSV-A incidence gradually increased reaching its highest prevalence in the final year of the study. Co-infections between RSV-A and RSV-B were detected during the periods of co-circulation (Figure 1).

### 3.3. Socio-demographic, clinical characteristics, and risk factors of RSV infection

In the current study, male predominance was noted with RSV infection (male: female = 1.7:1) and RSV-A infection (male: female = 2.8:1). The occurrence of RSV infection in the first year of life was high (73.77%) and RSV infection was more common in the first 6 months of life (41.46%). Age < 1 year was significantly associated with RSV, RSV-A and RSV-B infections (*p* < 0.05). Male sex was significantly associated with RSV-A infection (*p* < 0.05; Table 1). Based on our findings, RSV infection was mostly detected from the Sinhalese children (93.9%), the major ethnic group living in Sri Lanka. Most of the RSV infected patients resides in rural (53.04%) and urban (45.12%) areas in Kegalle. However, ethnicity and living area of the patients did not show any statistical significance (*p* > 0.05) with infections with RSV or RSV subtypes.

**Table 1:** Categorization of respiratory syncytial virus (RSV) infected children based on the sex and age (months).

Sex and age	Total ARTI *n* = 500; (%)	RSV Positive *n* = 164; *n* (%)	RSV-A positive *n* = 46; *n* (%)	RSV-B positive *n* = 101; *n* (%)	RSV-A & -B co-infection *n* = 17; *n* (%)
Male (M)	296 (59.2)	103 (62.8)	34 (73.9)ᶲ	58 (57.43)	11 (57.42)
Female (F)	204 (40.8)	61 (37.2)	12 (26.1)	43 (42.57)	6 (35.29)
M: F ratio	1.4: 1	1.7: 1	2.8: 1	1.3: 1	1.8: 1
Median age (months)	12.6	10.19	8.89	10.43	10.91
First year of life					
1–6 months	156 (31.2)	68 (41.46)	21 (45.65)	37 (36.63)	8 (47.05)
7–12 months	178 (35.6)	53 (32.31)	13 (28.26)	39 (38.61)	3 (17.4)
1–12 months	334 (66.8)	121 (73.77)ᶲ	34 (73.91)^#^	76 (75.24)^#^	11 (64.45)
Second year of life					
13–18 months	72 (14.4)	23 (14.02)	8 (17.39)	13 (12.87)	3 (17.64)
19–24 months	36 (7.2)	9 (5.48)	2 (4.37)	5 (4.9)	1 (5.88)
3 to 5 years of life					
25–36 months	35 (7.0)	7 (4.26)	0	3 (2.97)	0
37–48 months	12 (2.4)	3 (1.82)	0	3 (2.97)	0
49–60 months	11 (2.2)	1 (0.6)	0	1 (0.99)	0
>60 months	0	0	0	0	0

^*^Correlation is significant at *p* < 0.05 by Chi-square test; ^#^Correlation is significant at *p* < 0.05 by Kruskal-Wallis test; ^ᶲ^Correlation is significant at *p* < 0.05 by Binary logistic analysis.

Moderate bronchiolitis, fever for > 4 days, cough, headache, dyspnea, conjunctivitis, tachypnoea, diarrhea, fatigue, severe dehydration and stuffiness (blocked nose with mucus) were significantly associated with RSV infection in general (*p* < 0.05). Moderate bronchiolitis, fever for > 4 days, dyspnea and tachypnoea were significantly associated with RSV-A infection (*p* < 0.05). Mild bronchiolitis, moderate bronchiolitis, fever for > 4 days, headache, severe dehydration and cough were significantly associated with RSV-B infection (*p* < 0.05). Cold, stuffiness and nasal congestion were significantly associated with RSV-A and B co-infections (*p* < 0.05; Tables 2, 3). Number of people living at home (*n* > 6), inhaling toxic fumes and having pets at home were significantly associated with RSV infection (*p* < 0.05). Family history of asthma was significantly associated with RSV-A infection (*p* < 0.05). Having pets at home was significantly associated with RSV-B infection and RSV-A and-B co-infection (*p* < 0.05; Table 4).

**Table 2:** Clinical categorization of RSV-associated ARTI in children on admission to the hospital.

Clinical characteristics	RSV positive (*n* = 164)	RSV-A positive (*n* = 48)	RSV-B positive (*n* = 101)	RSV-A and B co-infection (*n* = 17)
Fever	132	34	83	15
Fever days >4	102^#^ᶲ	24^#^ᶲ	75^#^ᶲ	3
Cough	136*ᶲ	44	75*	17
Cold	133	34	93	16*
Shortness of breath	23	5	16	2
Difficulty in breathing	84	27	50	7
Wheezing	6	0	4	2
Headache	7*	4	3*	0
Vomiting	28	8	19	1
Runny nose	128	40	72	16
Dyspnea	49*	16*	28	5
Conjunctivitis	3*ᶲ	1	2	0
Tachypnoea	46*	16*	27	3
Nasal congestion	3	1	1	1^+^
Nasal block	8	2	5	1
Chills	16	5	10	1
Diarrhea	13*	5	7	1
Fatigue	4*ᶲ	0	4	0
Stiffness	7*ᶲ	1	4	2*ᶲ
Severe dehydration	*3^#^*	1	*2^#^*	0
High dependency care	1	1	0	0
Intensive Care	16	5	10	1

*Correlation is significant at *p* < 0.05 by Chi-square test; ^#^Correlation is significant at p < 0.05 by Kruskal–Wallis test; ᶲCorrelation is significant at *p* < 0.05 by binary logistic analysis.

**Table 3:** Clinical characteristics of RSV infected children in the study sample.

Clinical categorization	RSV positive *n* = 164; (%)	RSV-A positive *n* = 46; (%)	RSV-B positive *n* = 101; (%)	RSV-A and B co-infection *n* = 17; (%)
Mild bronchiolitis (Øymar et al., 2014)	32 (19.51)	5 (10.86)	26 (25.74)*	1 (5.88)
Moderate bronchiolitis	81 (49.39)*	30 (65.21)*	42 (41.58)*	9 (52.94)
Severe bronchiolitis	1 (0.60)	0	1 (0.99)	0
Persistent bronchiolitis	1 (0.60)	0	1 (0.99)	0
Bronchopneumonia	23 (14.02)	5 (10.86)	17 (16.83)	1 (5.88)
Unclassified lower respiratory tract infections	24 (14.63)	6 (13.04)	12 (11.88)	6 (35.29)
Upper respiratory tract infections	1 (0.60)	0	1 (0.99)	0
Right middle lobar pneumonia	1 (0.60)	0	1 (0.99)	0
Right side lobar pneumonia	0	0	0	0

* Correlation is significant at p < 0.05 by Chi-square test.

**Table 4:** Risk factors for acquiring RSV infection in children less than 5 years.

Risk factors	RSV positive *n* = 164; (%)	RSV-A positive *n* = 46; (%)	RSV-B positive *n* = 101; (%)	RSV-A and B co-infection *n* = 17; (%)
Overcrowding (> 6 family members in a house)	20 (12.19)ᶲ^#^	6 (13.04)	12 (11.88)	2 (11.76)
Family history of asthma	27 (16.46)	12 (26.08)*ᶲ	14 (13.86)	1 (5.88)
Cigarette smokers at home	25 (15.24)	8 (17.39)	15 (14.85)	2 (11.76)
Toxic fumes at home	113 (68.90)*ᶲ	31 (67.39)	72 (71.28)	10 (58.82)
Allergic to pollen dust	0	0	0	0
Recent long distance travel	8 (4.87)	3 (6.52)	5 (4.95)	0
Pets at home	54 (32.92)*ᶲ	15 (32.60)	32 (31.68)ᶲ	7 (41.17)ᶲ

*Correlation is significant at p < 0.05 by Chi-square test; ^#^ Correlation is significant at p < 0.05 by Kruskal-Wallis test; ᶲ Correlation is significant at p < 0.05 by Binary logistic analysis.

**Figure 2:**
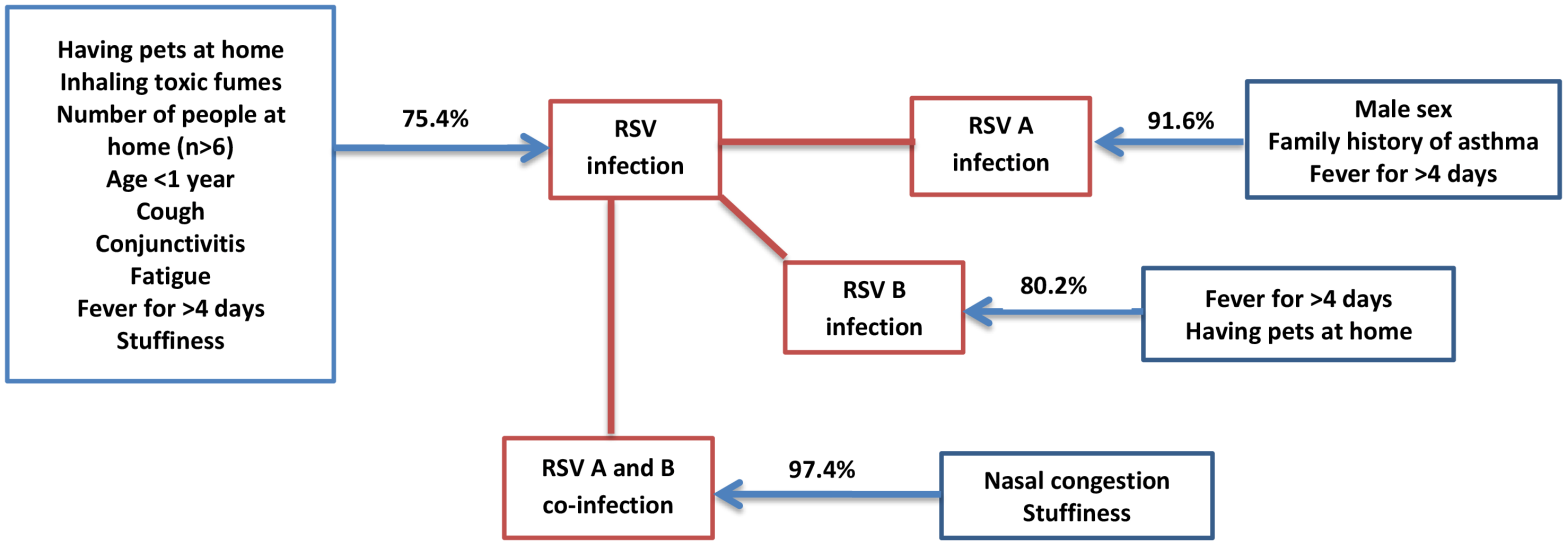
Predictive analysis of risk factors to get RSV infection. The predictive map developed using the data obtained from the study to identify how risk factors can be used to predict the probability (%) of getting infection with RSV, RSV-A, RSV-B and co-infections between RSV-A and-B in children <5 years in Sri Lanka.

Eventually, a predictive map was developed using binary regression analysis to identify the likelihood of developing infection with RSV, RSV-A, RSV-B and co-infection between RSV-A and RSV-B in children < 5 years based on risk factors. The clinical and socio-demographic risk factors like age < 1 year, fever for >4 days, cough, conjunctivitis, stuffiness, fatigue, number of people at home (*n* > 6), having pets at home and inhaling toxic fumes together have a probability of 75.4% to develop RSV infection in children < 5 years with ARTI in Sri Lanka. Likewise, male sex, fever for > 4 days and family history of asthma together have a probability 91.6% to develop RSV-A infection in children < 5 years with ARTI in Sri Lanka; fever for > 4 days and having pets at home together have a probability of 80.2% to develop RSV-B infection in children < 5 years with ARTI in Sri Lanka; stuffiness and nasal congestion together have a probability of 97.4% to develop RSV-A and RSV-B co-infection in children < 5 years with ARTI in Sri Lanka (Figure 2).

### 3.4. The impact of climatic factors for acquiring RSV infection

**Figure 3:**
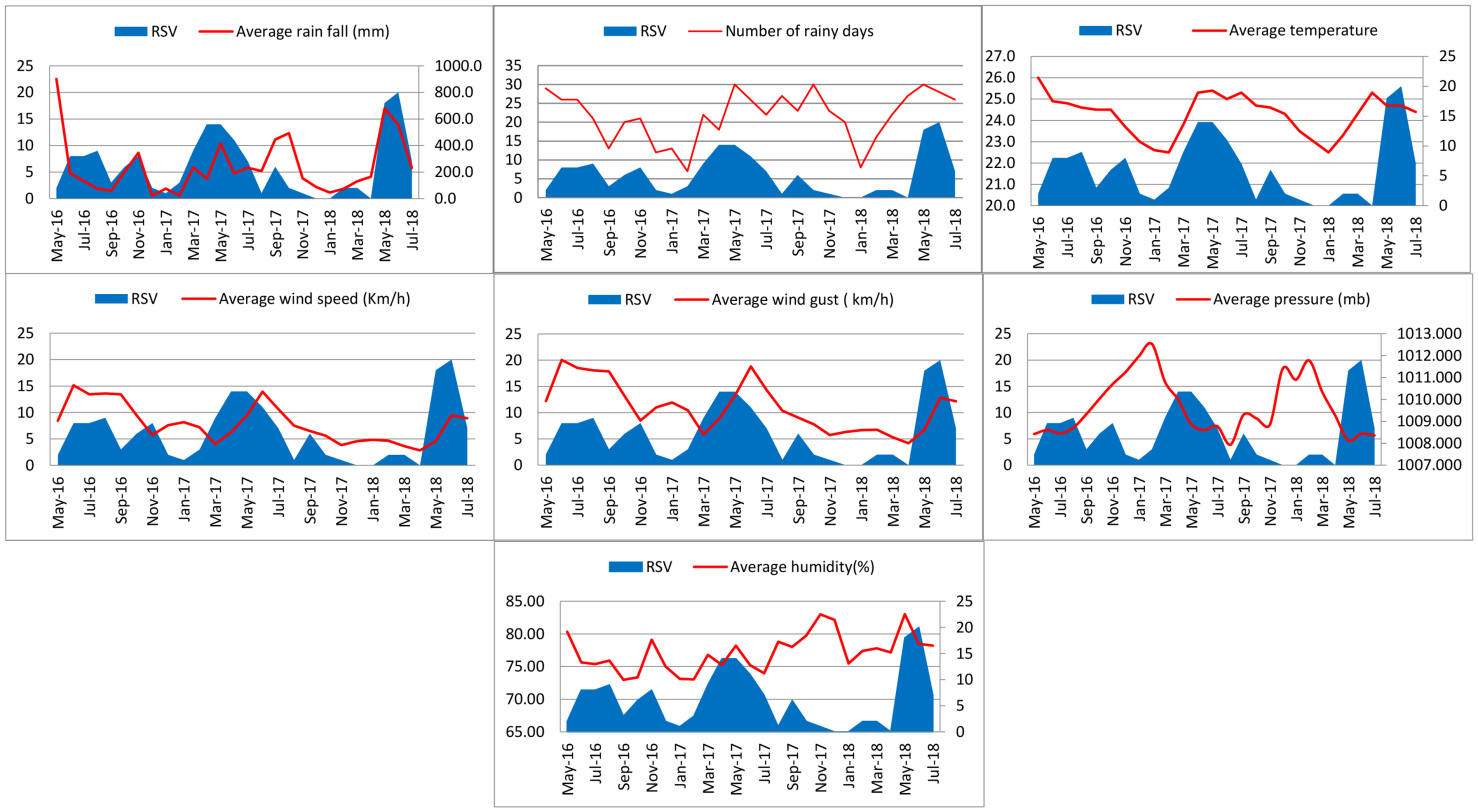
Monthly distribution of RSV infection with rain fall, number of rainy days, temperature, wind speed and gust, atmospheric pressure and humidity from May 2016 to July 2018.

RSV was detected throughout the year with three major peaks (June–August 2016, March–July 2017 and May–July 2018) and two minor peaks (October–November 2016 and September 2017) during the study period. The first RSV peak (June–August 2016) during the study occurred after the onset of a seasonal rainfall 2 months prior (May 2016). The second RSV outbreak (October–November 2016) occurred during the inter-monsoon lasting from September to December in 2016. The third RSV peak (March–July 2017) occurred during the rainy season from the south-west monsoon from March to October 2017. The final RSV peak (May–July 2018) occurred along with a period of high rainfall during the south-west monsoon lasting from March to July 2018. Only the last RSV peak (May–July 2018) reached its peak during the highest range of humidity. Temperature appeared to have a considerable impact on RSV activity as high temperatures reported during October 2016, April 2017 and April 2018 overlapping the first month of RSV peak activity during October to November in 2016, April to July in 2017 and May to July in 2018. Increase in wind speed (Km/h) and wind gust (Km/h) appeared simultaneously with every RSV peak during the study period. Increase in atmospheric pressure (mb) appeared to overlap the one major peak (March–July 2017) and one minor peak (October–November 2016; Figure 3).

In the current study, increases in temperature (°C), wind speed (Km/h) and wind gust (Km/h) showed a very strong correlation with the RSV infection in children (*p* value < 0.01). Increases in rainfall (mm) and atmospheric pressure (mb) showed a strong correlation with the RSV infection in children (*p* value = 0.05–0.01). RSV subtypes did not show any statistical correlation with any of the climatic factors.

## 4. Discussion

Based on the global estimates in 2019, RSV is responsible for more than 95% of ARTI episodes in children < years. Moreover, severe RSV-associated ARTI is a major cause of mortality in children and 97% of these deaths occur in developing countries (Li et al., 2022). Based on our study findings, RSV was the most common viral etiology associated with ARTI in the study population. RSV was detected throughout the study period with peak periods occurring in some months. RSV burden and seasonal patterns of the current study are in agreement with those reported in Sri Lanka (Jayaweera et al., 2021) and elsewhere (Weber et al., 1998; Bhandary and Boloor, 2016; Kim et al., 2020).

In our study, RSV-B was the most common RSV subtype detected although there was a year around circulation of both RSV types. RSV accounts for most co-infections with other respiratory viruses. Co-infections between RSV-A and RSV-B were detected during peak times of co-circulation. Based on previous studies, RSV-A has been detected as the commonest subtype circulating in many Asian countries (Kim et al., 2020; Luo et al., 2020; Jia et al., 2022; Zhang et al., 2022). However, RSV-B was the commonest subtype circulating in Sri Lanka based on our findings. It may be that the pattern of circulation of RSV subtypes may differ country to country with time.

Moderate bronchiolitis, fever for > 4 days, cough, headache, dyspnea, conjunctivitis, tachypnoea, diarrhea, fatigue, severe dehydration and stuffiness were significantly associated with RSV infection. Moreover, moderate bronchiolitis, fever for >4 days, dyspnea and tachypnoea were significantly associated with RSV-A infection. Mild bronchiolitis, moderate bronchiolitis, fever for > 4 days, headache, severe dehydration and cough were significantly associated with RSV-B infection and cold and stuffiness were significantly associated with RSV-A and B co-infection. These clinical characteristics are in agreement with other research findings of RSV infection in children (La Via et al., 1992). RSV is the most common cause of bronchiolitis in infants and young children. Patients with RSV-associated bronchiolitis usually present with 2–4 days of upper respiratory tract symptoms such as fever, cold, rhinorrhea, and congestion, followed by lower respiratory tract symptoms such as frequent cough, wheezing, and difficulty in breathing with dyspnea or tachypnoea (Shi et al., 2017; Smith et al., 2017). Moreover, development of symptoms like asthma and recurrent wheezing is significantly associated with RSV infection in children less than 6 months (Fauroux et al., 2017). However, recurrent wheezing and asthma was not prominently associated to infection with RSV/RSV sub types in our study. RSV infection in general caused severe ARTI in the majority of the children in our study and dyspnea, tachypnoea and stuffiness were significantly associated with RSV infection. Similarly, infection with RSV-A subtype appeared to cause severe disease among children in the study sample and dyspnea and tachypnoea were significantly associated with RSV-A infection. On the contrary, the number of children who required high dependency care with RSV-B infection (*n* = 10) is double the number of children who required high dependency care with RSV-A infection (*n* = 5), although the association did not show a statistical significance. Hence, RSV-A infection showed a stronger correlation with severe ARTI in children < 5 years compared to RSV-B infection and RSV-A and B co-infection. Infection with RSV in general and RSV-A subtype appeared to cause severe ARTI causing breathing difficulties (*p* < 0.05) indicating the chances for developing hypoxemia. These findings are in agreement with previous studies, which show RSV infections are significantly associated with hypoxemia and severe disease in hospitalized children with ARTI in developing countries (Thomas et al., 2017). In agreement with our findings, increased severity due to infection with RSV-A was evident in previous studies (Hall et al., 1990; Laham et al., 2017). In contrast, increased severity with RSV-B infection and absence of disease severity in infection with RSV subtypes are also reported in other studies (McIntosh et al., 1993; Panayiotou et al., 2014).

In our study, number of people at home (*n* > 6), inhaling toxic fumes and having pets at home were significantly associated with RSV infection. Moreover, family history of asthma was associated with RSV-A infection and having pets at home was associated with RSV-B and RSV-A and B co-infections. Likewise, in many studies, crowding at home with many individuals and siblings, inhaling toxic fumes like tobacco smoke and family history of asthma have a significant association with RSV-associated ARTI (Sigurs et al., 1984; Shi et al., 2015; Jayaweera et al., 2016). Furthermore, the current study findings are similar to those reported from other developing countries, as severe RSV disease is strongly associated with crowding and contact intensity with school aged siblings and other family members (Weber et al., 1999). One of the possible mechanisms behind crowding and severe RSV infection may be the increased exposure to the RSV inoculum with the close proximity of contact (Okiro et al., 2008). In developing countries, inhaling and exposure to toxic fumes are identified as risk factors for RSV-associated ARTI and ARTI overall (Weber et al., 1999; Shi et al., 2015). According to the WHO, half of the population in developing countries uses coal and biomass in the form of wood, dung and crop residues for domestic energy and these materials were burnt in simple stoves with incomplete combustion. Moreover, there is a strong correlation between poverty in developing countries and the use of polluting fuels which leads to air pollution. Consequently, woman and children who stay at home longer are exposed to high levels of indoor air pollution, making them at risk for RSV infection (Bruce et al., 2000). There is a lack of literature suggesting any direct association between having pets at home and RSV-associated ARTI in children. However, a few studies have pointed out that having pets like dogs at the time of RSV infection could increase the risk of asthma due to the combined effect of the virus and the allergen in increasing the allergic airway response (Siegle et al., 2010; Adamko and Friesen, 2012).

In our study, age < 1 year was significantly associated with infections with RSV/RSV subtypes and male predominance was significantly associated with RSV-A infection. In agreement with the current study, age < 1 year and male sex also have been reported by some other studies in association with RSV infection in children (Nagayama et al., 2006; Stein et al., 2017; Jayaweera et al., 2021). Although, early infancy (< 6 months) did not show any significant association with infections with RSV / RSV subtypes in the study sample, the number of RSV infected children in the first 6 month of age group was high compared to the second 6 months of age and this finding is in agreement with previous studies (Kaneko et al., 2002; Scheltema et al., 2017; Ueno et al., 2019; Andeweg et al., 2021). Increased prevalence of RSV infection in children in early infancy is believed to be related to waning of maternal antibodies. Moreover, high rate of RSV infection in the first 6 months of life indicates that the maternally derived immunity is not sufficient to prevent severe RSV infection in infants. Poor responsiveness of maternal antibodies may also contribute to high rate of RSV infection in the first 6 months of life and poor responsiveness of maternal antibodies is believed to be due to suppressive effects of epitope masking and phagocytosis of antibody–virus complexes (Ueno et al., 2019).

Male predominance in RSV infections is believed to be due to immuno-modulatory effect of male sex hormones during neonatal age making the boys more vulnerable to the infection (Nagayama et al., 2006). Additionally, increased physical activity in boys from infancy suggests increased motor activity making them more susceptible to the infection than girls through active engagement of boys with other individuals at home and in the community (Thomas and Thomas, 1988). In our study, infection with RSV/RSV subtypes did not show any statistical significance with ethnicity and residence. However, the association between RSV infection with ethnicity and residence has been reported by a few other studies in other countries and in Sri Lanka (Clarke et al., 1978; Bigogo et al., 2013; Kassem et al., 2019; Jayaweera et al., 2021). It is not clear whether there is a strong association between ethnicity and RSV infection in children (Kassem et al., 2019). On the other hand, there is a strong association between difficulties in accessing medical care for children with RSV infection in rural areas in developing countries and this shows the negative influence of rural living on severe RSV-associated ARTI (Weber et al., 2002).

We were able to produce a predictive map for RSV infection in children using clinical and socio-demographic risk factors and this will be useful to predict RSV infection in children solely based on the associated risk factors. According to the map developed using multiple binary logistic regression analysis, certain risk factors like age < 1 year, fever for > 4 days, cough, conjunctivitis, stuffiness, fatigue, number of people at home (*n* > 6), having pets at home and inhaling toxic fumes, showed a high probability of getting RSV infection in children < 5 years. The risk factors showing the probability of getting RSV-A infection in children < 5 years were male sex, fever for > 4 days and family history of asthma. The risk factors showing the probability of getting RSV-B infection in children < 5 years were fever for > 4 days and having pets at home. The risk factors showing the probability of getting RSV-A and B co-infections were stuffiness and nasal congestion. The map would help predict RSV/RSV subtype infection and RSV-A and B co-infection based on the presenting clinical and socio-demographic risk factors in children < 5 years with ARTI in developing countries. Pattern of clinical and socio-demographic risk for RSV infection has been reported by studies done elsewhere and Sri Lanka (Hall et al., 1990; La Via et al., 1992; Weber et al., 1999; Nagayama et al., 2006; Shi et al., 2015; Fauroux et al., 2017; Laham et al., 2017; Smith et al., 2017; Stein et al., 2017; Jayaweera et al., 2021).

Respiratory syncytial virus was prevalent throughout the year with peaks in certain months in our study period. Major RSV peaks were observed from June to August 2016, March to July 2017 and May to July 2018. RSV is the most common respiratory virus affecting children causing outbreaks all over the world despite the climate or geographical area. In most climates, RSV activity is present during the whole year or one half of the year with peak periods in certain months. RSV infection is seasonal in most countries and the seasonality varies considerably between regions. In tropical or subtropical climates, RSV outbreaks are predominantly associated with the rainy season and RSV activity usually peaks after the onset of the rains. In islands and countries closer to the equator with perennial high rainfall, RSV transmissions occur throughout the year or most part of the year (Weber et al., 1998). Conversely, high rate of RSV-associated mortality in children outside of the RSV season in tropical and subtropical countries suggests year around RSV activity in those regions (Shi et al., 2015). RSV activity positively correlated with increased rainfall and temperature in our study. It suggests that RSV seasonality in Sri Lanka has characteristics similar to tropical countries located closer to the equator with perennial high rainfall (Weber et al., 1998; Jayaweera et al., 2021). Moreover, it has been postulated that the increased rainfall and high temperatures are associated with stability and survival of the virus in harsh environments, which facilitate transmission (Thongpan et al., 2020). In our study, increased atmospheric pressure (barometric pressure) was correlated with RSV activity. According to another study, there is an association between increases in atmospheric pressure and RSV transmission (Hervás et al., 2012). It is likely that increase in the atmospheric pressure may result in a lower dispersion of RSV containing droplets when an infected individual expels them. As viral infectivity depends on the size of the viral inoculum, a low degree of dispersion of infective particles may produce a high concentration of infective virus facilitating the active transmission (Hervás et al., 2012). There was a strong correlation between increases in wind gust and wind speed with increased RSV activity, this has been reported by other studies as well (Hervás et al., 2012; Rodriguez-Martinez et al., 2015). Further research is needed to understand the influence of wind speed in the transmission of RSV.

Use of IFA for the preliminary screening as a limitation of our study, considering the lower sensitivity of the assay compared to the PCR. A large scale longitudinal study conducted over a longer period using real time PCR would identify the actual prevalence, seasonality and disease burden of RSV / RSV subtypes in the study population.

## 5. Conclusion

In the current study, RSV was the most predominant viral cause of childhood ARTI and hospitalization. RSV-B was the most common subtype circulated in the study sample. Co-infections among RSV subtypes and other viruses (Inf-A, Inf-B, PIV-1, PIV-2 and PIV-3) were also detected. During the study duration (2016–2018), RSV was prevalent throughout with peak periods in certain months. RSV-A and RSV-B co-circulated during the study period and co-infections between RSV-A and RSV-B were detected during the peak periods. RSV infection in general caused severe ARTI in the majority of the children. RSV-A infection caused severe disease leading to hypoxemia and many children infected with RSV-B required high dependency care. Co-infection between RSV-A and B did not increase the disease severity. In the current study, clinical and socio-demographic risk factors for RSV infection were moderate bronchiolitis, fever for >4 days, cough, headache, dyspnea, conjunctivitis, tachypnoea, diarrhea, fatigue, stuffiness, number of people living at home (n > 6), inhaling toxic fumes, age < 1 year and having pets at home. The predictive map shows a probability of 75.4% for contracting RSV infection, if a child has the following clinical and socio-demographic features collectively: age < 1 year, fever for > 4 days, cough, conjunctivitis, stuffiness, fatigue, number of people at home (*n* > 6), having pets at home and inhaling toxic fumes. In the current study, there was a strong correlation between increases in rainfall, temperature, wind gust, wind speed and atmospheric pressure with increased RSV activity. This study provides some important information on the epidemiology of RSV-associated ARTI in children less than 5 years of age and these findings would help implement prevention and control strategies against RSV infection in Sri Lanka.

## Data availability statement

The original contributions presented in the study are included in the article/supplementary material, further inquiries can be directed to the corresponding author.

## Ethics statement

The studies involving human participants were reviewed and approved by Ethical Review Committee, Faculty of Medicine, University of Peradeniya, Sri Lanka. Written informed consent to participate in this study was provided by the participants’ legal guardian/next of kin.

## Author contributions

MD collected the samples from the hospital, performed laboratory work including IFA and PCR, analyzed and interpreted the patient data regarding RSV infection, and wrote the original draft of the manuscript. RR collected the samples from the hospital and performed IFA. AM selected patients for the project based on a criteria, collected the samples from the patients, and co-supervised the project. CA did the statistical analysis using patient data. FN conceptualized the study, supervised the project, acquired funding, and reviewed and revised the manuscript. All authors contributed to the article and approved the submitted version.

## Funding

This work was supported by the National Science Foundation of Sri Lanka [Grant no: NSF/SCH/2017/01].

## Conflict of interest

The authors declare that the research was conducted in the absence of any commercial or financial relationships that could be construed as a potential conflict of interest.

## Publisher’s note

All claims expressed in this article are solely those of the authors and do not necessarily represent those of their affiliated organizations, or those of the publisher, the editors and the reviewers. Any product that may be evaluated in this article, or claim that may be made by its manufacturer, is not guaranteed or endorsed by the publisher.

## Abbreviations

RSV: respiratory syncytial virus
ARTI: acute respiratory tract infection
LMIC: low-income and middle-income countries
NPA: nasopharyngeal aspirate
IFA: immunofluorescence assay
PCR: polymerase chain reaction
Inf: Influenza
PIV: para influenza
